# Diabetes, Protein Misfolding, and Heat Stress: Molecular Insights and Translational Perspectives

**DOI:** 10.1055/a-2790-5251

**Published:** 2026-02-09

**Authors:** M. Marc Abreu, Victor Hugo Spitz, David M. Smadja

**Affiliations:** 1 Department of Clinical Sciences, BTT Medical Institute, Aventura, Florida, United States; 2 Department of Biomedical Engineering and Medical Physics, BTT Medical Institute, Aventura, Florida, United States; 3 Department of Medicine, Nova Southeastern University Medical School, Fort Lauderdale, Florida, United States; 4 Université Paris Cité, Institut national de la santé et de la recherche médicale (INSERM), Paris Cardiovascular Research Center (PARCC), Paris, France; 5 Hematology Department, Assistance Publique–Hôpitaux de Paris (AP–HP), European Georges Pompidou Hospital, Paris, France

**Keywords:** cardiovascular disease, heat shock proteins, heat therapy, hyperthermia, mitochondrial dysfunction, diabetes mellitus, endothelial progenitor cells, thrombosis, amylin

## Abstract

Diabetes mellitus (DM), particularly type 1 (T1D) and type 2 (T2D), is a growing global health burden with complex pathophysiology extending beyond glucose dysregulation. Both forms of diabetes involve cellular stress, chronic inflammation, and β-cell dysfunction. Heat shock proteins (HSPs), key mediators of protein homeostasis and immune modulation, are emerging as critical players in the progression and complications of diabetes. In T1D, HSPs act at the crossroads of β-cell stress and autoimmunity, while in T2D, their dysregulation contributes to insulin resistance and mitochondrial dysfunction. Misfolded proteins, particularly amylin aggregates, further drive β-cell apoptosis in T2D and may influence immune activation in T1D. Hyperthermia (HT) and passive heat therapy (hT) activate protective stress responses through HSP induction, mimicking some benefits of exercise and improving glycemic control, insulin sensitivity, and vascular health. Preclinical and early clinical studies suggest that thermal interventions could complement standard care, especially in patients unable to engage in regular physical activity. This review consolidates current evidence on the roles of HSPs, amylin misfolding, and hT in the pathogenesis of diabetes, with a particular emphasis on vascular and thrombotic complications and their therapeutic implications. Targeting these converging molecular pathways may offer new avenues for preserving β-cell function, mitigating metabolic complications, and enhancing diabetes management.

## Introduction

Diabetes mellitus (DM) remains one of the most significant cardiovascular diseases and chronic metabolic disorders worldwide, with escalating prevalence due to global lifestyle shifts. Diabetes 1 2 represents a harmful systemic environment that disrupts the processes of revascularization and tissue repair following ischemia 3 4 but is also a significant risk factor in the progression of aortic valve stenosis and calcification. 5 Metabolic disorders and chronic hyperglycemia impair endothelial cells and increase the accumulation of advanced glycation end products (AGEs), which accelerate calcification. 5 6 7 8 Since diabetic patients experience strong vascular disorders and faster disease progression and poorer outcomes after valve replacement, understanding diabetes and vascular or valvular disease interplay may lead to targeted therapeutic strategies. 5 Among its most common types, type 1 diabetes (T1D) is primarily characterized by autoimmune destruction of pancreatic β-cells, resulting in absolute insulin deficiency, whereas type 2 diabetes (T2D) involves insulin resistance and relative insulin deficiency. Beyond these canonical definitions, emerging research highlights complex molecular and cellular mechanisms that underlie disease onset and progression in both T1D and T2D. 9 10

Recent findings suggest that heat shock proteins (HSPs) play a pivotal role in the development of β-cell impairment, insulin resistance, and a range of diabetes-related complications, including both microvascular and macrovascular dysfunctions. 11 12 In particular, HSP70 exerts protective effects on cells by mitigating oxidative stress, dampening inflammatory responses, preventing programmed cell death, and facilitating the repair and proper folding of damaged proteins. 13 In recent years, a compelling body of evidence has focused on the role of misfolded proteins, specifically islet amyloid polypeptide (IAPP, also known as amylin), 14 15 and the protective function of HSPs as crucial modulators in diabetes. 16 17 Additionally, novel interventions such as hyperthermia (HT) and heat therapy (hT) have been explored as potential therapeutic strategies. 18 19 In particular, passive hT, such as sauna bathing and hot water immersion, has been traditionally used in various cultures and is known to improve cardiovascular health and glucose metabolism in non-diabetic individuals. 20 21 Emerging evidence suggests that hT might activate similar metabolic and anti-inflammatory pathways as exercise, potentially enhancing insulin sensitivity and supporting glycemic control in patients with T2D. 22 The convergence of these pathways offers a complex and nuanced perspective on diabetes that goes beyond the traditional focus on glucose metabolism, incorporating immunological aspects, protein homeostasis mechanisms, and cellular stress responses.

This article aims to synthesize and critically examine the roles of misfolded proteins and HSPs in both T1D and T2D, with a particular focus on their contribution to vascular and thrombotic complications. Furthermore, it evaluates the potential of HT and hT as emerging interventions, drawing on recent findings from both preclinical and clinical studies.

## Methods

This narrative review was developed through a structured literature search of peer-reviewed studies published between January 2000 and June 2025. We searched PubMed, Web of Science, and Scopus using combinations of keywords including “heat shock proteins,” “HSP,” “hyperthermia,” “heat therapy,” “diabetes,” “insulin resistance,” “vascular complications,” and “amylin.” Only articles in English and involving either human or animal models were included. Clinical trials, mechanistic studies, systematic reviews, and meta-analyses were prioritized. The reference list was managed using EndNote to ensure consistency and eliminate duplication.

## Heat Shock Proteins and Type 1 Diabetes

HSPs, highly conserved molecular chaperones, play a fundamental role in maintaining cellular homeostasis, particularly under stress conditions. In the context of T1D, HSPs are increasingly recognized as major pathophysiology factors, pivotal immunological modulators acting at the interface of β-cell stress, autoimmunity, and inflammation 17 ( Table 1 ). The central role of HSPs in both cellular protection and immune recognition makes them key contributors to the pathogenesis and progression of T1D.

**Table 1:** Differential roles of heat shock proteins in type 1 and type 2 diabetes pathogenesis

HSPs involvement in T1D		References
HSP27	In individuals recently diagnosed with fulminant or acute-onset T1D, presence of autoantibodies targeting HSP10 has been observed.	52 134 162
DNAJ family/HSP40	Patients exhibited increased urinary levels of HSP40 when normalized to creatinine concentration.	163
HSP60	In NOD mice, HSP60 was detected on the surface of pancreatic β-cells prior to the onset of islet inflammation. This endogenous protein may serve as an autoantigen recognized by β-cells in the early stages of immune activation.	24 164 165
HSP70	Impaired ability to upregulate HSP70 under stress conditions contributed to increase inflammatory environment targeting pancreatic β-cells. Moreover, elevated levels of IgA antibodies directed against HSP70 indicated a specific humoral autoimmune response. Finally, HSP70 was found to associate with a key antigenic region of proinsulin, enhancing its uptake and presentation by antigen-presenting cells. Under inflammatory stress, GRP78 translocates to the surface of human β-cells via the secretory pathway—mediated by DNAJC3—where it acts as a signaling receptor that triggers proapoptotic pathways, forming a self-destructive loop distinct from its antiapoptotic role in cancer, and highlighting its potential as a therapeutic target in type 1 diabetes.	40 45 166 167 168
HSP90	Increased IgG1 and IgG3 autoantibodies targeting HSP90. Increased HSP90 concentrations are observed in young individuals exhibiting β-cell autoimmunity; however, these levels do not reliably differentiate between those who will or will not develop type 1 diabetes—whether in youth or adults.	47 169
HSPs involvement in T2D		References
HSP27	Suppressing the phosphorylation of IKK-β inducing insulin signaling, while also protecting β-cells from apoptosis by attenuating NF-κB activation and reducing caspase-3 enzymatic activity.	170 171
DNAJ family/HSP40	β-cell integrity is maintained in part by limiting eIF2α phosphorylation, which reduces endoplasmic reticulum (ER) stress and prevents translational overload. Loss of DNAJB3 function has been linked to heightened β-cell vulnerability due to the mitochondrial activation of proapoptotic Bcl-2 family members. Within the ER, DNAJB9 (also known as Erdj4) supports insulin responsiveness by restraining IRE1 signaling and lowering SREBP1c expression. In the mitochondrial context, DNAJC15 contributes to insulin resistance by suppressing complex I activity in the electron transport chain and impeding supercomplex assembly. Meanwhile, in the nuclear compartment, DNAJC27 influences metabolic regulation by promoting inflammatory signaling cascades, thereby disrupting insulin signaling dynamics.	172
HSP60	HSP60 contributes to β-cell preservation by counteracting hypertrophic effects induced by advanced glycation end products (AGEs) and also prevents insulin resistance and β-cell dysfunction by reducing oxidative stress. HSP60 also decreases proinflammatory cytokine production allowing modulation of mitochondrial double-stranded RNA release. Under normoglycemic conditions, cellular survival is maintained through direct interaction of specific proteins with Bax, preventing its mitochondrial translocation and the initiation of apoptosis. However, during hyperglycemia, excessive O-GlcNAcylation of HSP60 disrupts this interaction, facilitating Bax-mediated apoptosis in β-cells. Extracellular HSP60 contributes to the development of insulin resistance by engaging TLR4 within adipose and skeletal muscle tissues.	26 173 174
HSP70	The HSP70 family plays diverse roles in metabolic regulation and cell survival. iHSP72, located in the cytosol and nucleus, reduces inflammation and insulin resistance by blocking JNK/IKK signaling and protects β-cells from apoptosis by interfering with apoptotic proteins and ER stress pathways. Its extracellular form, eHSP72, promotes inflammation and β-cell dysfunction through TLR1/2 activation. GRP75 (mitochondrial) influences insulin sensitivity by altering mitochondrial complexes but can trigger β-cell death when overexpressed. GRP78, in the ER, impairs insulin signaling via SKIP interaction yet supports β-cell survival by managing amyloid stress. GRP170 enhances insulin signaling in liver and muscle cells.	57
HSP90	HSP90α may enhance insulin signaling by modulating nitric oxide levels, while HSP90β reduces glucose utilization by stabilizing PDK4 expression. Moreover, Hsp90α as a glucose-regulated surface protein that, through interaction with annexin II, may contribute to diabetes-related vascular complications by promoting plasmin activity and potential clotting abnormalities.	73 74 75

Abbreviations: HSP, heat shock protein; iHSP, intracellular heat shock protein; NOD, non-obese diabetic; TLR, Toll-like receptor; T1D, type 1 diabetes; T2D, type 2 diabetes.

This table summarizes key findings on the involvement of various HSP families in the pathophysiology of T1D and T2D. It highlights their roles in autoimmunity, insulin signaling, β-cell survival, and stress response mechanisms.

HSP60, one of the most studied chaperones, has been linked to the initiation and propagation of islet autoimmunity. HSP60 has been identified as a β-cell autoantigen in non-obese diabetic (NOD) mice and in humans with T1D. 23 24 It localizes predominantly in the mitochondria but also translocates to other cellular compartments during stress, which may render it immunogenic. 25 26 Furthermore, altered expression or posttranslational modifications of HSP60 can lead to the presentation of novel epitopes, triggering autoreactive T-cell responses. 27 28 Studies have demonstrated that in individuals with T1D, there is an increased presence of autoantibodies and autoreactive T cells specific for HSP60 and its derived peptides. 29 30 Notably, the peptide p277 from HSP60 has been shown to shift immune responses from a Th1 to a Th2 phenotype, thereby modulating autoimmune responses in NOD mice and human clinical trials. 31 32 This immune deviation is consistent with observations that T1D patients exhibit altered cytokine responses to HSP60. 29 33 Recent meta-analyses have indicated that despite initial promise, certain clinical trials were withdrawn, and antigen-specific immunotherapies have failed to demonstrate efficacy in halting the advancement of autoimmune diabetes in individuals with a new diagnosis. 34

The major histocompatibility complex (MHC) region encodes several HSPs, which are co-located with classical immune genes. 35 Polymorphisms in MHC-linked HSP genes have been associated with susceptibility to T1D. 36 These proteins, when aberrantly expressed or secreted during β-cell stress, may serve as danger-associated molecular patterns (DAMPs), further stimulating the innate immune system. 37

HSP70, another critical player, is upregulated in pancreatic islets in response to cytokines and oxidative stress. 38 Its intracellular role involves protecting β-cells from nitric oxide and free radical-mediated damage. 39 Paradoxically, extracellular HSP70 can be immunostimulatory, activating dendritic cells and inducing proinflammatory cytokine release. 40 Elevated HSP70 levels have been documented in the serum and urine of T1D patients, and these increases correlate with disease activity and complications. 41 Exosomal pathways serve as conduits for HSP secretion. Studies indicate that β-cells under inflammatory stress release exosomes containing HSPs along with autoantigens such as glutamic acid decarboxylase 65-kDa isoform (GAD65) and proinsulin, potentially priming autoreactive lymphocytes. 42 This process may facilitate antigen cross-presentation and perpetuate autoimmunity. Additionally, HSP90, often secreted under similar conditions, has emerged as a marker of β-cell stress and may enhance antigen presentation by stabilizing peptide–MHC complexes. 43 Endoplasmic reticulum (ER) stress is a critical component of β-cell dysfunction in T1D, and HSPs are integral to managing this stress. Glucose-regulated protein 78/binding immunoglobulin protein (GRP78 (BiP)), a key ER chaperone, is translocated to the plasma membrane under cytokine stress, triggering apoptotic signaling in β-cells. 44 From a systems perspective, T1D may reflect a failure of immunological tolerance due to chronic inflammation and insufficient stress adaptation. HSPs act not only as molecular buffers but also as signaling molecules in immune networks. Their extracellular release—whether passive via necrosis or active via vesicle export—functions as an alarm system to alert immune cells to danger. 37 This dual role—protective within the cell and immunogenic outside—places HSPs at a unique crossroads of self-recognition and immune activation. Antibodies to HSPs, particularly HSP60 and HSP70, have been detected in various autoimmune and metabolic contexts. Elevated IgA responses to HSP70 correlate with vascular complications in diabetes, while anti-HSP60 titers are predictive of atherosclerotic risk. 45 46 In T1D, these antibodies may represent not only diagnostic biomarkers but also reflect ongoing tissue injury and stress. 47 Moreover, the broader stress response system, including HSPs, interfaces with metabolic regulators and redox signaling. HSPs modulate antioxidant responses, and their upregulation is protective in models of oxidative β-cell injury. 39 Their dysfunction may thus compromise β-cell survival under immune assault. Emerging research supports the concept that enhancing HSP expression or function may bolster cellular resistance and immune tolerance, offering novel avenues for preserving β-cell mass in at-risk individuals.

Finally, T1D continues to rise globally and remains a serious condition despite advances in treatment. 1 48 HSPs are implicated in disease onset, acting both as immune triggers and modulators. They can provoke autoimmune T-cell responses but also promote regulatory mechanisms that suppress inflammation. This duality makes HSPs promising candidates for combination therapies aimed at preserving β-cell function. Additionally, their early expression patterns suggest they may serve as useful biomarkers for disease prediction and prevention.

## Heat Shock Proteins and Type 2 Diabetes

T2D is a complex metabolic disorder defined by insulin resistance, impaired insulin signaling, chronic inflammation, and β-cell dysfunction. 49 Central to the progression of this disease is the imbalance between cellular stress and the body's protective mechanisms ( Table 1 ). Among these protective responses, HSPs serve as molecular chaperones that ensure protein stability and folding, and are crucial for maintaining cellular homeostasis under stress conditions. 16 In the context of T2D, their role is increasingly recognized as pivotal, not only in protein maintenance but also in modulating inflammatory pathways that influence insulin sensitivity. HSPs, particularly HSP60, HSP70, HSP72, and HSP90, are produced in response to various stressors, including hyperglycemia, oxidative stress, and inflammation, and participate in the pathophysiology of T2D and also its vascular complications. 50 51 52 53 They are involved in critical processes, such as refolding misfolded proteins, preventing protein aggregation, and guiding proteins toward degradation pathways when irreparable damage occurs. Under normal circumstances, these functions maintain cellular integrity and protect against apoptosis. However, in the chronically inflamed and metabolically overloaded environment characteristic of T2D, the function of HSPs becomes altered, contributing to disease progression. 16 54 One of the central stress responses activated during metabolic overload is the unfolded protein response, triggered by the accumulation of misfolded proteins in the ER. HSPs are key mediators of the unfolded protein response and help restore ER function. Yet, persistent ER stress can overwhelm this adaptive mechanism, resulting in the activation of inflammatory signaling cascades, such as the NF-κB pathway, which elevates levels of proinflammatory cytokines like IL-1β, TNF-α, and IL-6. 55 56 57 These cytokines further exacerbate insulin resistance by interfering with insulin receptor signaling. Extracellular HSPs also act as immune modulators. When secreted or released from damaged cells, HSPs such as HSP60 and HSP70 can function as DAMPs, activating pattern recognition receptors, such as Toll-like receptors (TLRs). For instance, HSP60 has been shown to engage TLR2 and TLR4, stimulating immune cells to release inflammatory cytokines, thereby contributing to the systemic inflammation observed in T2D patients. 58 59 Similarly, extracellular HSP70 can activate antigen-presenting cells, enhancing the inflammatory response, despite having cytoprotective functions when retained intracellularly. 60 61 In adipose tissue, a central organ in metabolic regulation, HSPs appear to mediate the inflammatory state associated with obesity-induced insulin resistance. Studies have revealed that intracellular levels of HSP70 are reduced in adipose tissue of obese and diabetic individuals, coinciding with elevated cytokine expression and macrophage infiltration. 62 63 Moreover, Chung et al demonstrated that individuals with obesity and insulin resistance exhibit reduced levels of HSP72 in skeletal muscle. 64 This decrease in HSP70 is significant because the inducible form, HSP72, has been shown to inhibit stress kinases like JNK, which are known to impair insulin signaling through the serine phosphorylation of insulin receptor substrate-1 (IRS-1). 64 Conversely, overexpression of HSP72 in skeletal muscle improves insulin sensitivity and glucose uptake, highlighting its potential as a protective factor in metabolic tissues. 65 The use of pharmacological inducers of HSP expression further underscores their importance in T2D pathogenesis and therapy. BGP-15, a hydroxylamine derivative, has demonstrated the capacity to elevate HSP70 levels in various tissues. 66 67 68 Indeed, BGP-15 enhances insulin sensitivity in various insulin-resistant animal models and in human clinical trials. Its effect is comparable to rosiglitazone, highlighting its therapeutic potential for T2D. 69 70 71 Finally, HSP90α may enhance insulin signaling by modulating nitric oxide levels, while HSP90β reduces glucose utilization by stabilizing pyruvate dehydrogenase kinase 4 (PDK4) expression. 72 Hsp90α, as a glucose-regulated surface protein, through interaction with annexin II, may contribute to diabetes-related vascular complications by promoting plasmin activity and potential clotting abnormalities. 73 74

Mitochondrial function is another key area where HSPs exert influence. HSP60 and HSP10 are mitochondrial chaperonins that protect against oxidative stress and help maintain mitochondrial integrity. Their reduced expression in insulin-resistant states correlates with decreased oxidative phosphorylation capacity, especially in skeletal muscle and liver tissues, which are central to systemic glucose homeostasis. 75 HSPs are also implicated in immune system interactions beyond their role in innate signaling. Chronic inflammation, a hallmark of T2D, is perpetuated by the recruitment and activation of immune cells in metabolic tissues. HSPs contribute to the polarization and activation of these immune cells, directly influencing cytokine production. For example, HSP70 can act on monocytes and dendritic cells to promote a proinflammatory phenotype, reinforcing the vicious cycle between metabolic dysfunction and immune activation. 61 Importantly, lifestyle interventions such as physical exercise are potent inducers of HSP expression. Endurance and resistance training have both been shown to increase HSP72 levels in skeletal muscle, correlating with reductions in inflammatory markers and improvements in insulin sensitivity. 53 62 76 In individuals with T2D, the heat shock response is often blunted, and restoring this through regular exercise yields metabolic improvements. Similarly, dietary antioxidants like curcumin, resveratrol, and α-lipoic acid have been shown to induce HSPs and possess anti-inflammatory effects, offering another avenue for non-pharmacological intervention. 77 78 Given their multifaceted roles in metabolic regulation, inflammation, and immune signaling, HSPs are being explored as both biomarkers and therapeutic targets in T2D. Circulating levels of HSP60 and HSP70 have been proposed as indicators of disease activity, although interpretation remains complex due to the dual nature of HSPs depending on their intracellular versus extracellular localization. 59 75 77 79 80 Other HSPs have also been shown to be regulated in diabetes, such as HSP47. 81 HSP47 has been previously identified as a stress-inducible collagen-binding chaperone, 12 and recent evidence suggests it plays a critical role in the retinal complications of diabetes. 81 In retinal Müller cells, high glucose induces HSP47 expression, which enhances its interaction with inositol-requiring enzyme 1 alpha (IRE1α), activating the IRE1α/spliced X-box binding protein 1 (XBP1-s)/hypoxia-inducible factor 1 alpha (HIF-1α) signaling pathway. Silencing HSP47 disrupts this pathway, reducing cytokine expression, suggesting a key role for HSP47 in diabetic retinal inflammation. 81 Moreover, in diabetic nephropathy (DN), HSP47 has emerged as a key player due to its role in promoting collagen synthesis, a major contributor to renal fibrosis and loss of function. HSP47 is overexpressed in human and experimental DN, particularly in mesangial cells, podocytes, and tubular epithelial cells, and co-localizes with increased type III and IV collagen. 52 82 Its induction is linked to TGF-β1 signaling and AGE accumulation. 52 While inhibition of HSP47 has shown promise in reducing fibrosis in experimental kidney disease models, 83 84 concerns remain due to impaired autophagy in DN, where HSP47 deletion may lead to procollagen accumulation and cell death. 85 Additionally, reduced HSP47 expression has been observed in diabetic wounds, suggesting that therapies targeting HSP47 could impair wound healing. 86 87

**Fig. 1:**
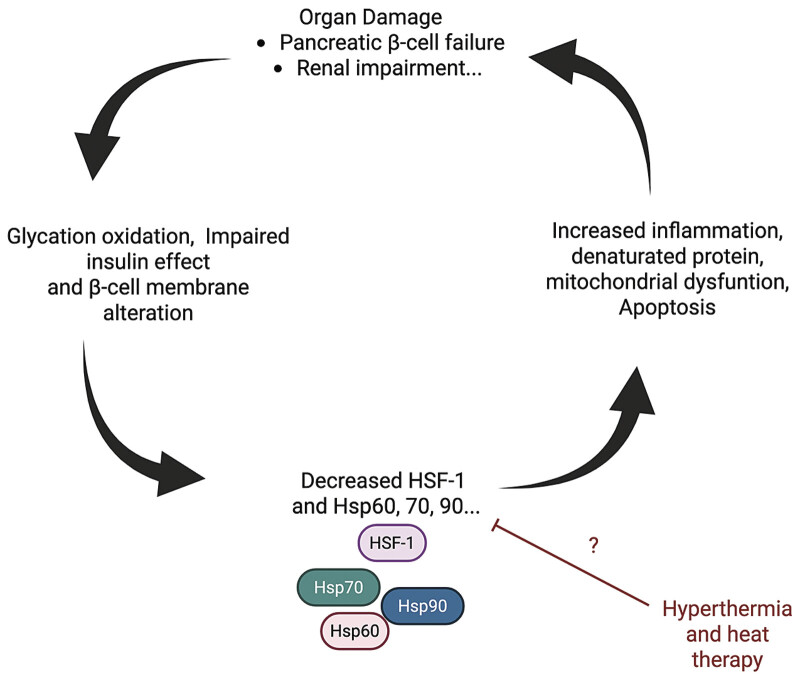
Conceptual model illustrating the pathological cascade linking type 2 diabetes and progressive organ damage. In type 2 diabetes, chronic metabolic stress triggers mitochondrial impairment, which leads to increased reactive oxygen species (ROS) production and cellular damage. This contributes to β-cell dysfunction and/or systemic inflammation create a self-reinforcing cycle of dysfunction that accelerates complications such as nephropathy. Heat therapy or hyperthermia may interrupt this pathological sequence by upregulating heat shock proteins (HSPs). Created with BioRender.com and licensed for journal publication. Created in BioRender. Smadja, D. (2025) https://BioRender.com/39b4n3w .

Finally, HSPs are deeply embedded in the molecular landscape of T2D ( Fig. 1 ). Their ability to modulate protein homeostasis, cellular stress responses, and immune activation places them at the intersection of inflammation and insulin resistance. Modifying the heat shock response through pharmacological agents, exercise, or dietary compounds presents a viable approach to improving metabolic health. Further research into tissue-specific HSPs regulation and delivery methods may unlock new pathways for managing and potentially preventing T2D.

## Amylin and Type 1 and Type 2 Diabetes

**Fig. 2:**
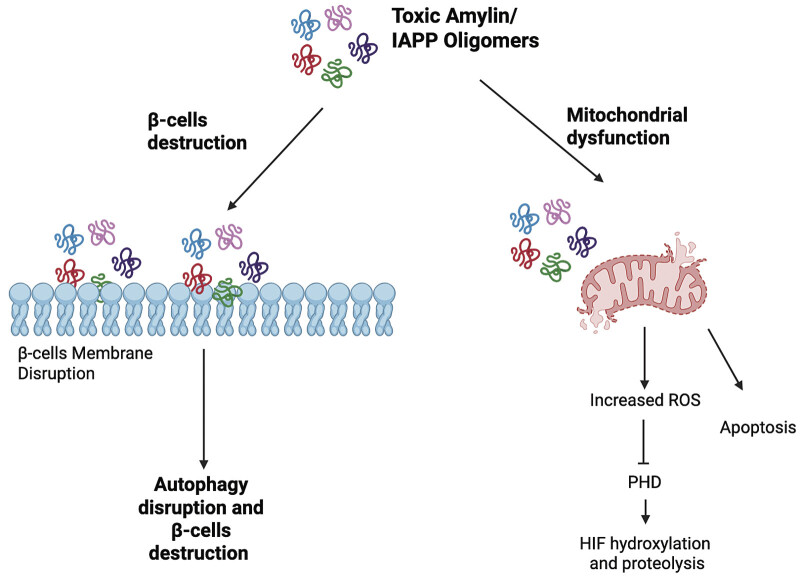
Toxic amylin oligomer accumulation drives pancreatic β-cell and mitochondrial dysfunction. Elevated levels of oligomeric human islet amyloid polypeptide (IAPP) contribute to pancreatic β-cell stress by disrupting mitochondrial function, impairing membrane integrity, promoting oxidative stress, and ultimately leading to β-cell dysfunction and apoptosis. This mechanism is a key contributor to β-cell failure in type 2 diabetes. Created with BioRender.com and licensed for journal publication. Created in BioRender. Smadja, D. (2025) https://BioRender.com/jz3cfy7 . ROS, reactive oxygen species.

A 37-residue peptide known as IAPP, also referred to as amylin, is released alongside insulin from the β-cells of the pancreas. 14 Under physiological conditions, amylin plays a crucial role in glycemic control by slowing gastric emptying, suppressing glucagon secretion, and promoting satiety. 14 Amylin is not a chaperone however, in pathological conditions (such as T2D), amylin forms toxic amyloid aggregates that are involved in the degeneration of pancreatic β-cells and mitochondrial dysfunction ( Fig. 2 ). In humans, amylin interacts with at least three distinct receptor complexes—AMY1, AMY2, and AMY3—each formed by the association of the calcitonin receptor with a specific receptor activity-modifying protein (RAMP). Under physiological conditions, amylin exists in a soluble, monomeric state. However, under certain conditions, individual amylin molecules may misfold, adopting conformations rich in β-sheet structures. These misfolded monomers tend to cluster, forming small oligomeric assemblies. As aggregation progresses, these oligomers can develop into protofibrillar intermediates—elongated and structurally unstable forms—that ultimately mature into stable amyloid fibrils.

In T2D, one of the hallmark pathological features is the misfolding and aggregation of amylin into amyloid fibrils, leading to plaque formation within the islets. These amyloid deposits disrupt islet structure, interfere with intercellular signaling, and cause direct cytotoxic effects on pancreatic β-cells. When pancreatic β-cells overproduce amylin, the accumulation can result in β-cell toxicity and destruction. 88 Insulin resistance, a hallmark of T2D, leads to chronic hyperglycemia and hyperinsulinemia. 89 This condition negatively impacts amyloid processing in the brain and in the pancreas while also stimulating mitochondrial oxidative stress. Though co-released with insulin, circulating amylin levels represent just 1% to 2% of insulin concentrations and increase in response to elevated glucose. 90 91 Amylin is primarily degraded by insulin-degrading enzyme and neprilysin, both zinc-dependent proteases that also play a role in amyloid-β clearance, helping mitigate potential cytotoxic effects. 92 93 94 Aggregation of amylin in pancreatic islets is strongly associated with β-cell apoptosis and disease progression in T2D. 95 Paradoxically, in models of chemically induced diabetes, amylin administration improved β-cell viability by enhancing microvascular flow and modulating membrane polarization. 96

Beyond their direct cytotoxic effects on pancreatic β-cells, mounting evidence indicates that mitochondria are a principal target of amylin oligomers. 97 98 99 The functional integrity of these organelles is crucial for maintaining cellular energy homeostasis, redox balance, and survival. Indeed, amylin oligomers localize to mitochondria and induce membrane permeabilization, triggering the activation of apoptotic pathways. 100 Subsequent studies have confirmed that exposure to oligomeric amylin leads to the collapse of mitochondrial membrane potential and a marked increase in reactive oxygen species, indicative of oxidative stress. 100 101 These mechanisms collectively contribute to β-cell apoptosis and insulin deficiency. Importantly, the consequences of mitochondrial dysfunction extend beyond pancreatic islets. In the diabetic kidney, sustained mitochondrial impairment exacerbates tissue hypoxia, thereby amplifying oxidative damage and accelerating the progression of DN. 101 Thus, mitochondrial health represents a central axis linking β-cell failure, systemic metabolic stress, and end-organ complications in type 2 diabetes mellitus (T2DM).

Interestingly, while amyloid deposits are rare in T1D, there is evidence suggesting that misfolded amylin may contribute to β-cell stress and potentially to neoantigen formation. 102 103 Inflammatory environments prevalent in T1D can exacerbate amylin misfolding, potentially accelerating β-cell dysfunction. Additionally, the functional role of amylin deficiency in T1D remains an area of active research. Loss of amylin due to β-cell destruction can exacerbate glycemic variability and postprandial hyperglycemia. The introduction of pramlintide, a synthetic amylin analog, has demonstrated clinical benefits in improving postprandial glucose control and reducing insulin requirements, suggesting a potential therapeutic avenue. 14 The dual nature of amylin—as both a regulator of glucose metabolism and a source of toxic aggregates—reflects the fine balance required in its expression and processing. Future therapies may focus on modulating amylin levels to preserve its physiological functions while preventing pathological aggregation.

A case–control study examining plasma amylin levels found that concentrations were lower than insulin in healthy individuals, increased with obesity, were nearly absent from T1D, and showed variable trends in T2D—suggesting that amylin may participate in glucose homeostasis alongside insulin. 104

## Heat, Hyperthermia, and Diabetes Treatment

Initial clinical trials in T2D suggest that hT may offer metabolic benefits, particularly in improving glycemic control. In T2D, hT interventions have consistently shown improvements in insulin sensitivity and glucose tolerance. Sebők et al conducted a systematic review and meta-analysis that consolidated evidence from human and animal studies, highlighting slight yet consistent improvements in glycemic control with hT. 22 The analysis included five studies comprising diverse thermal interventions, such as hot water immersion and sauna therapy. Overall, passive heating was associated with modest improvements in fasting plasma glucose and glycated hemoglobin (HbA1c) levels, though the changes did not always reach statistical significance. The weighted mean difference for HbA1c was −0.55%, suggesting a clinically meaningful, albeit small, benefit. While the effect size is limited, it parallels benefits seen with some lifestyle interventions and warrants further exploration. The benefits are thought to arise from improved endothelial function, enhanced insulin receptor signaling, and reductions in chronic low-grade inflammation. Notably, the systemic nature of hT allows for concurrent targeting of multiple tissues, including skeletal muscle, adipose tissue, and the liver, which are central to glucose homeostasis. While these findings are promising, translation to clinical practice requires careful consideration of safety, optimal thermal dosing, and individual patient characteristics. Future studies should aim to define standardized protocols and evaluate long-term outcomes in diverse diabetic populations. Future randomized controlled trials with standardized protocols, larger cohorts, and extended follow-up are essential to confirm these preliminary findings. Nevertheless, hT represents a novel, non-pharmacological intervention with the potential to complement existing lifestyle and pharmacologic treatments for T2D.

To better understand these clinical observations and guide future therapeutic applications, it is essential to explore the underlying mechanisms through which hT exerts its metabolic benefits—particularly those related to the heat shock response and HSP-mediated cellular adaptations demonstrated in both preclinical and emerging human studies. Recent scientific advances increasingly highlight hT as a promising alternative or complementary strategy to traditional physical exercise for improving metabolic health, particularly in individuals with T2D. Indeed, thermal exposure—such as through sauna use or immersion in hot water—represents a classic method for triggering the heat shock response (HSR) and stimulating the expression of HSPs. 105 This novel approach is especially relevant given the growing prevalence of sedentary lifestyles and the rising incidence of obesity and T2DM worldwide. While regular exercise remains a cornerstone intervention for improving insulin sensitivity and enhancing mitochondrial function, many patients encounter barriers to physical activity due to aging, chronic pain, musculoskeletal limitations, neurological impairments, or cognitive decline. 106 In this context, hT—including modalities such as sauna bathing, hot water immersion (e.g., hot tubs), and heated wraps—emerges as a feasible and practical option to stimulate similar physiological benefits.

There are several preclinical proofs of the beneficial effects of HT or hT on diabetes. In aged rodent models, applying HT twice weekly for 30 minutes over an 8-week period led to a marked upregulation of HSP70 expression in muscle tissue—doubling within 24 hours after a single session and tripling by the study's conclusion. The intervention also yielded metabolic benefits, including reduced HbA1c, enhanced insulin receptor signaling, and lower fasting insulin levels. 107 Similar outcomes were reported in low-density lipoprotein (LDL) receptor-deficient mice on a high-fat diet, where 8 weeks of weekly heat exposure elevated HSP72, HSP73, and HSP27 in endothelial cells of the aorta. This molecular response was accompanied by reductions in total cholesterol, triglycerides, LDL cholesterol (LDL-C), fasting glucose, insulin resistance, and oxidative stress, along with increased high-density lipoprotein (HDL) cholesterol. 108 In another rodent study, diabetic rats induced via streptozotocin and subjected to hot water immersion (42°C, 30 minutes, five times a week for 3 weeks) showed higher circulating HSP60 levels. 109

Despite these promising findings in animal models, human research on HT's capacity to modulate both intracellular and extracellular HSPs expression remains limited. One study involving 14 lean and overweight individuals undergoing 60 minutes of passive heat exposure at 40°C reported elevated serum levels of HSP70 and IL-6 immediately following intervention. These increases coincided with higher energy expenditure and reduced postprandial glucose peaks. 110 However, the effects of HT on intracellular HSP (iHSP) expression in humans are less consistent. Some research has demonstrated iHSP72 upregulation in peripheral blood mononuclear cells (PBMCs) after 8 weeks of 60-minute hot water immersion at 40.5°C, 111 112 and increased iHSP72 in skeletal muscle following 6 days of 2-hour localized heating via pulsed wave diathermy. 113 Conversely, another study with overweight male participants found that while 2 weeks of brief hot water immersion (39°C for 10 minutes) reduced fasting glucose, it did not significantly alter iHSP levels in monocytes. 114

Heat exposure has been shown to activate molecular pathways that promote metabolic health, particularly through the induction of HSPs. The historical foundation for using heat as a therapeutic intervention in diabetes can be traced back to the work of Hooper, published in the *New England Journal of Medicine* , 115 who first reported that hot-tub therapy could lower HbA1c levels and improve overall glycemic control in patients with T2DM. 115 Building on this pioneering observation, Chung et al demonstrated that individuals with obesity and insulin resistance exhibit reduced levels of HSP72 in skeletal muscle. 64 Their study revealed that hT increases HSP72 expression, and when overexpressed in animal models, it protects against insulin resistance induced by high-fat diets. 64 Further supporting these findings, Gupte and colleagues conducted studies in rodents fed a high-fat diet and found that hT significantly improved glucose tolerance, enhanced skeletal muscle insulin sensitivity, and robustly induced the heat shock response. 116 Pallubinsky et al extended this evidence to humans by demonstrating that 10 days of passive heat exposure in overweight individuals led to improvements in glucose metabolism and increased fat oxidation, even in the absence of measurable changes in iHSP72 levels. 117 Such improvements may be partially mediated by the ability of hT to modulate insulin action through HSP regulation, suppress chronic low-grade inflammation, and enhance mitochondrial bioenergetics. 57 Interestingly, the effects of heat exposure differ depending on the duration and frequency of application. Acute heat exposure may transiently impair mitochondrial function due to increased mitochondrial fission; however, repeated treatments—applied daily or several times per week—promote mitochondrial remodeling, increase phosphorylation efficiency, and enhance overall respiratory capacity. 118 These adaptations are crucial because mitochondrial dysfunction is a key feature of metabolic diseases and contributes to insulin resistance and impaired glucose utilization. Furthermore, Laukkanen and Kunutsor provided a detailed analysis of heat therapy protocols, particularly focusing on Finnish sauna bathing and hot water immersion. 20 Their comprehensive review emphasized the broad spectrum of health benefits associated with regular passive hT, including improvements in cardiovascular health, enhanced endothelial function, and reduced systemic inflammation. 20 This evidence suggests that incorporating hT into lifestyle interventions may offer a viable and effective strategy for extending health span and managing chronic metabolic diseases.

Given that a substantial proportion of individuals with T2D or obesity are unable to perform regular physical activity due to physical limitations, acute injuries, or severe fatigue, hT stands out as an accessible and low-risk intervention. It offers a unique combination of metabolic, cardiovascular, and potentially cognitive benefits without the musculoskeletal strain or safety risks sometimes associated with exercise interventions. 106 As the global burden of metabolic diseases continues to grow, integrating heat-based modalities into clinical practice may provide an essential adjunct to traditional therapeutic strategies, ultimately improving patient outcomes and quality of life.

HT, or therapeutic heating, has emerged as an innovative strategy for modulating metabolic and inflammatory pathways involved in diabetes pathophysiology. 57 It has garnered special attention for individuals with T2DM who often face limitations to regular exercise due to obesity, musculoskeletal disorders, frailty, or other comorbidities. 20 Preclinical studies have provided strong support for the metabolic advantages of HT. For example, Kokura et al investigated the effects of whole-body hyperthermia (WBH) in obese diabetic mice and demonstrated significant reductions in fasting blood glucose levels after repeated sessions. 119 Additionally, WBH-treated mice showed improved insulin sensitivity, as indicated by enhanced glucose and insulin tolerance test results. 119 At the molecular level, skeletal muscle from these mice exhibited increased expression of glucose transporter type 4 (GLUT4), reflecting enhanced glucose uptake capacity in peripheral tissues. 119 Moreover, HT has been suggested to mimic several physiological effects typically induced by exercise, such as increased peripheral blood (PB) flow, enhanced mitochondrial efficiency, and upregulation of glucose transport mechanisms. 117 Exercise is widely known to improve insulin sensitivity through mechanisms independent of weight loss, and HT might provide a comparable metabolic stimulus, making it an attractive alternative or adjunct for patients unable to engage in sufficient physical activity. 106 Beyond metabolic regulation, HT has shown potent anti-inflammatory and antioxidative effects in preclinical models of diabetes. Chronic low-grade inflammation and elevated oxidative stress are major contributors to insulin resistance, endothelial dysfunction, and the progression of diabetic complications, including nephropathy and retinopathy. 120 Notably, WBH has been shown to reduce systemic inflammatory markers and oxidative damage, while also decreasing urinary albumin excretion in diabetic mice, indicating potential renoprotective effects. 119 121 Furthermore, HT may positively influence lipid metabolism, modulate adipokine profiles, and increase energy expenditure, all of which can support long-term metabolic health. 21 Repeated heat exposure is also thought to promote mitochondrial remodeling and improve mitochondrial respiratory efficiency, crucial adaptations for maintaining cellular energy balance and metabolic flexibility. 118 Overall, these findings suggest that HT could serve as a valuable adjunctive therapy for diabetes management by leveraging endogenous protective mechanisms to improve glucose control, reduce inflammation, and potentially slow the progression of diabetes-related complications. 57 64 117 While promising, more clinical studies in humans are essential to validate these effects, define optimal treatment protocols, and ensure safety and feasibility for widespread use. 122 Beyond its effects on metabolic parameters, HT has shown immunomodulatory properties relevant to both T1D and T2D. In the context of T1D, fever-range WBH has demonstrated preventive effects in NOD mice. Capitano's team reported that weekly WBH sessions prevented the onset of T1D in NOD mice by enhancing natural killer (NK) cell activity and reducing pancreatic islet infiltration by autoreactive lymphocytes. 123 124

**Fig. 3:**
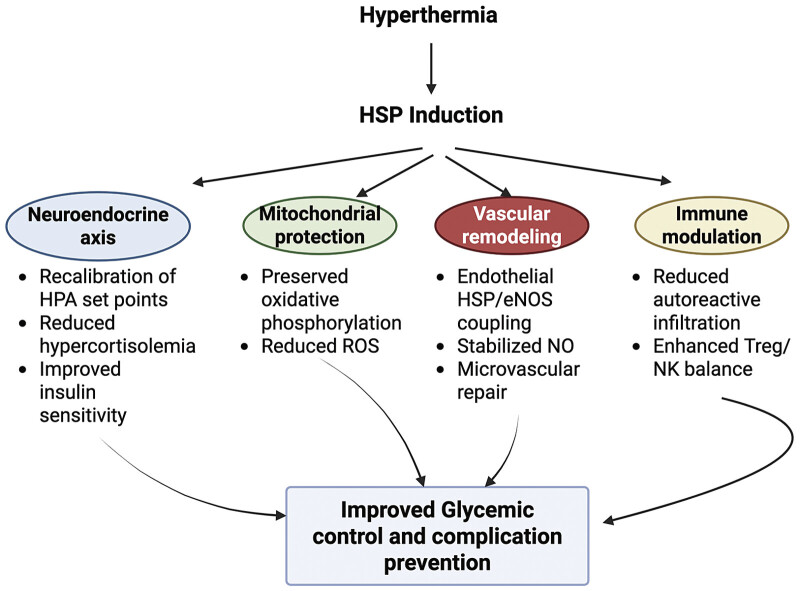
Mechanisms of heat therapy-induced glycemic control via heat shock protein (HSP) pathways. This diagram illustrates the proposed pathways through which heat therapy (HT) induces HSP expression, leading to improved glycemic control and prevention of complications. HSP induction influences four major physiological systems: (1) The neuroendocrine axis, by recalibrating HPA set points and improving insulin sensitivity; (2) mitochondrial protection, through preserved oxidative phosphorylation and reduced reactive oxygen species (ROS); (3) vascular remodeling, enhancing endothelial function and microvascular repair; and (4) immune modulation, via reduced autoreactive infiltration and balanced NK/Treg activity. Created with BioRender.com and licensed for journal publication. Created in BioRender. Smadja, D. (2025) https://BioRender.com/7rhsuf6 . NK, natural killer.

Literature supports the idea that fever-range HT recalibrates hypothalamic set points, modulates sympathetic output, and reduces chronic hypercortisolemia, 125 126 thus improving insulin sensitivity and decreasing β-cell strain. Thermal activation of hypothalamic nuclei also normalizes hepatic gluconeogenesis and enhances β-cell responsiveness via vagal efferents, providing a neuroendocrine mechanism for improved fasting glycemia and postprandial insulin dynamics. The recalibration of central thermoregulatory and neuroimmune circuits links thermal therapy with pancreatic and hepatic outcomes, 127 128 reinforcing a brain-centered approach to diabetes treatment, especially within the whole-body hyperthermia–brain-targeted therapy (WBH-BTT) framework. 129 130 Beyond autonomic control, thermal stimulation of hypothalamic and limbic networks may engage the neuroimmune interface, transiently activating microglial and astroglial HSP responses that propagate anti-inflammatory signaling to peripheral organs. At the cellular level, HT induces HSP70/72, which helps preserve mitochondrial integrity by preventing permeability transition, enhancing oxidative phosphorylation, and limiting reactive oxygen species (ROS) generation in β-cells and kidneys. Repeated HSP70/72 induction may also confer a “proteostasis memory,” reinforcing chaperone networks and epigenetically stabilizing metabolic genes; such adaptive imprinting likely underlies sustained benefits seen with recurrent hyperthermic exposure in animals and humans. Parallel findings from exercise physiology and thermal conditioning studies suggest that recurrent subthreshold heat stress induces cross-tolerance, enhancing resilience against metabolic and oxidative insults. These actions may interrupt hyperglycemia-related organelle stress, slowing the development of nephropathy and neuropathy. 131 132 Concomitantly, HT appears to normalize adipokine profiles—lowering leptin and resistin while restoring adiponectin secretion—which complements HSP-mediated improvements in insulin signaling and mitochondrial efficiency. In the vasculature, thermal preconditioning upregulates endothelial HSPs, boosts endothelial nitric oxide synthase (eNOS) coupling, and stabilizes nitric oxide (NO) bioavailability—key factors in preventing microvascular damage like retinal capillary dropout or renal ischemia. 133 134 Furthermore, in T1D models, HT promotes immune tolerance by suppressing autoreactive T-cell infiltration in islets and enhancing NK cell activity and regulatory T-cell function, potentially reshaping the immune landscape to preserve β-cell function. 135 136 In terms of dose–response, literature on therapeutic thermal hormesis indicates that controlled exposure (∼39.5–41.5°C) for approximately 60 minutes can robustly activate HSP responses, akin to exercise-induced stress resilience. 137 138 Future studies should correlate thermal dose with mechanistic and clinical readouts—circulating/exosomal HSP70, IL-6/C-reactive protein (CRP), endothelial function (flow-mediated dilation [FMD]), and continuous glucose monitoring (CGM) metrics (time-in-range, glycemic variability) to anchor outcomes to the proposed pathways. Finally, thermal preconditioning upregulates endothelial HSPs, enhances eNOS coupling, and stabilizes NO bioavailability—countering microvascular rarefaction. 139 140 Chronic HT could theoretically improve perfusion in the retina, kidneys, and peripheral nerves, altering the trajectory of complication development in this patient population. 141 142 Taken together, coordinated neuroendocrine, mitochondrial, vascular, and immune adaptations converge to restore metabolic flexibility and slow progression of diabetic complications. Looking ahead, integrating standardized WBH protocols with real-time thermal monitoring could make HT safer for patients with autonomic neuropathy or heart failure, enhancing scalability and reproducibility across clinics. By simultaneously targeting neuroendocrine regulation, mitochondrial stability, vascular homeostasis, and immune tolerance, HT represents a unified systems-medicine strategy capable of addressing both metabolic dysfunction and its downstream complications ( Fig. 3 ).

## Vascular and Thrombotic Complications of Diabetes: Endothelial Stress and Repair Imbalance

DM markedly increases the risk of both microvascular (retinopathy, nephropathy) and macrovascular (myocardial infarction [MI], stroke) complications, primarily through chronic injury to the vascular endothelium. This endothelial dysfunction results from a dual imbalance: An overactivation of cellular stress pathways, particularly via HSPs, and a loss of vascular repair capacity, potentially due to the depletion of endothelial progenitor cells (EPCs). 143 144 In patients with T2D and lower limb ischemia, Rabczynski et al found that anti-HSP60/65 antibodies were elevated and significantly correlated with von Willebrand factor (vWF), a marker of endothelial damage, indicating a potential autoimmune mechanism contributing to endothelial dysfunction in diabetic macroangiopathy. 145 Although the antibody levels were not significantly higher than in controls, their association with vWF suggests that immune responses against HSPs may exacerbate vascular injury in diabetes through chronic inflammatory and prothrombotic pathways.

Among HSPs, HSP47 plays a central role in promoting thrombosis and fibrosis. 12 It facilitates platelet–collagen binding and stabilizes thrombus formation, and is overexpressed in diabetic conditions. Its inhibition has been shown to reduce thrombus size and platelet aggregation without affecting thrombin signaling. 146 Beyond its thrombotic role, HSP47 is also a key mediator of endothelial-to-mesenchymal transition in cardiac endothelial cells under stressors such as aging, obesity, and pressure overload. This transition is driven by TGF-β and oxidative stress and results in endothelial senescence and dysfunction—processes reversed by exercise, which downregulates HSP47 and restores vascular homeostasis. 147 Complementing this, Khalil et al demonstrated that HSP47 plays a critical role in cardiac fibrosis. 148 Using cell-specific gene deletion models, they showed that only deletion of HSP47 in myofibroblasts—not in cardiomyocytes or endothelial cells—was effective in blocking collagen deposition, reducing cardiac hypertrophy, and limiting fibrosis after injury. Endothelial-specific deletion of HSP47 mildly reduced collagen type I but did not alter overall fibrosis, indicating that endothelial cells contribute minimally to fibrotic remodeling in the heart. In parallel, other HSPs contribute to endothelial pathology in diabetes. HSP60, elevated in diabetic states, activates TLRs and NF-κB, driving chronic vascular inflammation. While improved glycemic control reduces systemic IL-6 levels, anti-HSP60 antibodies often remain elevated, suggesting persistent immune-mediated endothelial activation. 149 These stress-mediated pathways are not limited to large vessels. In the retina, HSP27 and HSP70 help counter oxidative stress, while in the kidney, HSP dysregulation is linked to podocyte–endothelial cross-talk and progression of DN. 52 Moreover, hyperglycemia-induced O-GlcNAcylation of key signaling proteins, such as Akt and eNOS, further impairs endothelial responses and reduces HSP72, a cytoprotective chaperone. These effects have been shown to be reversible with renin–angiotensin–aldosterone system (RAAS) inhibition, which may exert part of its renoprotective action through modulation of O-linked β-N-acetylglucosamine [a post-translational protein modification] (O-GlcNAc) signaling. 150 In the retina, HSP47 also plays a critical inflammatory role. Under high-glucose conditions, it activates the IRE1α/XBP1s/HIF-1α ER stress pathway in Müller glial cells, promoting production of VEGF, PDGF-B, and inducible nitric oxide synthase (iNOS). Silencing HSP47 blunts this inflammatory cascade, revealing its potential as a cross-organ mediator of vascular inflammation and fibrosis. 81 While HSPs amplify stress and senescence in diabetic vasculature, the loss of EPCs undermines the system's capacity for repair. EPCs, especially CD34 ^+^ subsets, are critical for vascular regeneration. 144 151 Diabetes reduces their number and function, with lower circulating levels predicting progression of renal disease and albuminuria. 152 Importantly, EPC-based therapies are emerging as a potential intervention to restore vascular health in diabetes, 153 154 155 in particular, because of their anti-inflammatory properties. 156 157 In preclinical models of DN and cardiomyopathy, EPC transplantation enhances microvascular density, reduces fibrosis, and improves both cardiac and renal function. These reparative effects are mediated via both direct incorporation and paracrine signaling, including the release of protective exosomes enriched in HSP20, p-Akt, superoxide dismutase 1 (SOD1), and survivin. 158 159 Further complicating vascular pathology in diabetes, bone turnover markers like osteocalcin, procollagen type I N-terminal propeptide (PINP) and C-terminal telopeptide of type I collagen (CTX) are independently associated with intracranial and extracranial artery stenosis. These markers, tightly connected to HSP-regulated stress responses, correlate with HbA1c and LDL-C levels and may serve as biomarkers for vascular calcification and instability. 160 Salybekov et al provide pivotal insights into how DM disrupts immune and vascular repair responses following MI, through a detailed murine model of T2D. 161 Their study identifies profound dysfunction in the cardio–spleno–bone marrow axis, leading to both impaired immune cell mobilization and EPC activity. Despite normal immune cell production in bone marrow, diabetic mice exhibited reduced PB levels of CD45 ^+^ leukocytes, neutrophils, and macrophages, particularly during the acute phase (days 1–3 post-MI), indicating ineffective mobilization. Notably, neutrophil polarization shifted from proinflammatory (N1) to anti-inflammatory (N2) types in the spleen, while macrophage recruitment, especially of the reparative M2 subset, was delayed and diminished in diabetic hearts. In the adaptive immune compartment, CD8 ^+^ cytotoxic T cells predominated in diabetic hearts across all time points, contrasting with CD4 ^+^ helper T-cell dominance in controls, a pattern that may exacerbate inflammation and cardiac injury. B-cell infiltration and PB levels were also significantly suppressed in diabetic mice during early MI stages, suggesting impaired humoral immune involvement. Crucially, EPC-colony-forming capacity was markedly reduced in diabetic animals across the bone marrow, spleen, and PB compartments. The shift toward primitive EPC colonies over definitive, mature ones suggests a differentiation block under diabetic stress, with implications for compromised neovascularization and repair. These findings align with earlier clinical observations of reduced EPC mobilization in diabetic MI patients and underscore the synergistic impact of immune dysregulation and vascular repair failure in diabetic ischemic injury. This study complements the broader understanding that hyperglycemia-induced stress, immune imbalance, and EPC dysfunction form a “double-hit” mechanism that drives diabetic vascular complications, including endothelial senescence, thrombosis, and fibrotic remodeling across organ systems. 161

In summary, diabetic vasculopathy stems from a converging imbalance between heightened endothelial stress—driven by the overexpression of HSPs—and a diminished capacity for vascular repair due to EPC depletion. This dysregulation promotes endothelial senescence, thrombosis, chronic inflammation, and fibrotic remodeling across key organs such as the retina, heart, and kidneys. Addressing this pathological interplay, through modulation of HSP-mediated stress responses or restoration of EPC function, offers a promising avenue for the prevention or attenuation of vascular complications in diabetes.

## Conclusion and Future Perspectives

All in all, the interplay of misfolded proteins, amylin aggregation, ER stress, and inflammatory pathways underscores the complexity of diabetes beyond simple glucose dysregulation. Despite growing interest in thermal interventions, several limitations must be acknowledged. Human data remain limited and heterogeneous, with substantial variation in heat exposure duration, intensity, modality (e.g., sauna, hot water immersion, WBH), and outcome measures. Few studies use standardized temperature protocols or track long-term glycemic outcomes. Additionally, individual responses may be influenced by comorbidities, age, and fitness level. These gaps highlight the need for rigorous, multicenter, randomized controlled trials to clarify optimal dosing and clinical applications. Misfolded proteins and HSPs in general contribute significantly to β-cell dysfunction and death in both T1D and T2D, offering potential targets for therapeutic intervention. Amylin's dual roles—essential for glycemic regulation yet pathogenic when aggregated—highlight the need for nuanced approaches that preserve physiological functions while preventing toxic misfolding. The protective roles of HSPs, particularly HSP70, further emphasize the importance of cellular stress responses in diabetes pathophysiology. Strategies to enhance HSP expression may offer cytoprotective benefits, modulate immune responses, and improve insulin sensitivity. Emerging evidence supports the beneficial effects of HT and hT in both preclinical and clinical settings. These interventions harness endogenous stress response pathways to improve glucose metabolism, enhance insulin sensitivity, and modulate immune function. However, translation to clinical practice requires rigorous validation, careful patient selection, and standardized thermal protocols. The integration of HT-based therapies with conventional treatments and lifestyle modifications represents a promising frontier in diabetes management. Moving forward, research should focus on elucidating the precise molecular mechanisms underlying thermal interventions, optimizing dosing regimens, and evaluating long-term outcomes. In conclusion, the convergence of protein misfolding, amylin biology, HSP function, and HT interventions provides a rich landscape for innovative diabetes therapies. By addressing the disease at multiple molecular and cellular levels, these approaches hold the potential to transform diabetes care and improve patient outcomes.
